# Female teneral mating in a monandrous species

**DOI:** 10.1002/ece3.264

**Published:** 2012-07

**Authors:** Karine Monceau, Joan van Baaren

**Affiliations:** 1 INRA, UMR 1065 Santé et Agroécologie du VignobleISVV, F-33883 Villenave d’Ornon, France; 2 Université de Bordeaux, UMR 1065 Santé et Agroécologie du VignobleBordeaux Sciences Agro, F-33883 Villenave d’Ornon, France; 3 UMR CNRS 6553 ECOBIO, Equipe PaysaClim, Université Rennes 1Rennes, France

**Keywords:** Cockroaches, monandry, *Schultesia nitor*, sexual conflict, sexual maturity

## Abstract

*Schultesia nitor* is a gregarious species living in *Cacicus* and *Psarocolius* ssp. pouch-like nests. Due to gregariousness, opportunities for multiple copulations in both sexes are not supposed to be restricted. Females produce only one brood during their life and die within a few days following the birth of their nymphs, but this unique brood could be the result of either single or multiple mating events (i.e., monandry vs. polyandry). In this study, we first determined the age of sexual receptivity of both males and females. Larval development in this species is shorter in males than in females and thus, this species is protandric. Males were not able to copulate the day after emergence. Contrary to males, teneral females (i.e., females achieving their imaginal molt but not yet fully sclerotised and colored) were attractive and were able to mate with males. In the second experiment, we tested the existence of multiple matings in both sexes. Our results showed that females were monandrous whereas males were polygynous. Since we had observed that females were monoandrous, we expected them to be choosy and we determined their ability to discriminate between virgin and nonvirgin males. When given the choice, females preferred virgin males and overall, they were more successful at mating than experienced ones. Our results suggest that monandry may be primarily driven by the female’s short life-span fecundity. The occurrence of teneral mating in this species calls into question the existence of a male strategy for monopolizing females, and as well as the implication of female choice. Although further work is required, this species provides an interesting model for understanding sexual conflicts.

## Introduction

Due to anisogamy, females are considered as being the limiting sex whereas males compete to access reproduction. Differential investment of both sexes in reproduction usually results in divergent fitness interests, thus generating sexual conflicts (Stockley 1997; Chapman et al. 2003; Arnqvist and Rowe 2005; Wedell et al. 2006; Hosken et al. 2009).

In females, although one or few matings are sufficient enough to fertilize all their ovocytes, female multiple mating occurs in many species (Jennions and Petrie 2000; Zeh and Zeh 2001). It was shown that females maximize their fitness through this strategy (Arnqvist and Nilsson 2000; Hosken and Stockley 2003). For example, females can gain genetic benefits for their offspring by remating (Yasui 1998). However, multiple mating can also be detrimental to female fitness in increasing time and energy costs, predation rates, physical injuries, and parasite transmission probabilities (Arnqvist and Nilsson 2000, and references therein). Inversely, males performed multiple mating to increase their fitness. However, males multiplying copulations can also suffer a cost associated with sperm replenishment and thus, multiple copulations can reduce their life span (Dewsbury 1982; Wedell et al. 2002a; Oliver and Cordero 2009). To reduce such costs, males can limit the size of their ejaculate but consequently, females can experience sperm limitation if they are not able to remate (Wedell et al. 2002a). For example, females in *Nauphoeta cinerea* (Blattaria: Blaberidae) are able to discriminate between males based on their previous mating experience in order to limit costs associated with mating with sperm-depleted males (Harris and Moore 2005).

Basically, mating rates often appear to be below the optimal mating rate for females due to male manipulation of female mating behavior, that is, sexual conflicts (Arnqvist and Nilsson 2000). Males can ensure mating by adopting several strategies (Wedell et al. 2006) by providing benefits to females and offspring, by exploiting female sensory bias through seduction, or by making mating less costly than resistance. Therefore, balance between one, few, or many matings depends mainly on the trade-off between the benefits and costs associated with mating a female could expect. These trade-offs also involve other traits like survival or dispersal (Stearns 1976, 1989, 2000; Nylin and Gotthard 1998; Gasser et al. 2000; Braby 2002; Hernaman and Munday 2005). For example, reduced adult survival is balanced by earlier reproduction attempts and faster development to reach maturity (Gasser et al. 2000; Haugen 2000). In insects, female mating rates have also been shown to be influenced by factors which directly affect costs and benefits of multiple matings, such as food availability and quality (Gwynne 1990; Rowe et al. 1996; Torres-Vila et al. 2005; Fox and Moya-Laraño 2009), or habitats (Corley and Fjerdingstad, 2011; El-Niweiri and Moritz 2011). For example, in *Lasius niger* (Hymenoptera: Formicidae), queens in southern Europe perform multiple matings whereas queens from northern regions mate only once, southern regions being biotically richer than northern ones (Gaston 2000; Corley and Fjerdingstad 2011). In *Apis mellifera jemenitica* (Hymenoptera: Apidae), queen mating frequencies are negatively correlated with rainfalls (El-Niweiri and Moritz 2011).

Among the gradient of mating rates, only one mating during its lifetime (i.e., monandry) is considered as a female strategy, rare in insects (Arnqvist and Nilsson 2000; Hosken et al. 2009). This mating system is widespread in eusocial and parasitic solitary Hymenopteran (Ridley 1993; Boomsma and Ratnieks 1996; Strassmann 2001; Kronauer et al. 2011), but it has also been reported in several Dipteran (Gillies 1956; Goma 1963; Mahmood and Reisen 1980; Reisen et al. 1984; Yuval and Fritz 1994; Jones 2001; South and Arnqvist 2008), Lepidopteran (Svärd and Wiklund 1989; Wedell et al. 2002b), and Blattodean (Livingstone and Ramani 1978; Moore and Moore 2001; Jayakumar et al. 2002; Lihoreau and Rivault 2010). Although monandry in social Hymenopteran has been determined to advantage the evolution of eusociality (Strassmann 2001), single mating is poorly understood in other species in regards to the benefits provided by multiple matings (Arnqvist and Nilsson 2000; Wiklund et al. 2001; Wedell et al. 2002b). Nevertheless, several hypotheses have been proposed. First, monandry can be maintained in populations through male enforcement by controlling female remating behaviors if it gains fitness benefit from female unique mating (see Hosken et al. 2009 for a review). For example, males can transfer mating plugs with antiaphrodisiac compounds during copulation which render females unattractive for other potential partners (e.g., *Pieris* species, Lepidoptera: Pieridae; Andersson et al. 2000, 2003, 2004), or they can induce nonreceptivity through mechanical processes during copulation (e.g., *N. cinerea*; Roth 1962, 1964a). Monandry can also arise from abiotic constraints. In *Pieris napi* for instance, females exhibit two mating strategies: they can either be polyandrous or monandrous. Although these differences are under genetic control (Wedell et al. 2002b), monandry in this species has been shown to be selected in populations facing unfavorable weather conditions (Välimäki et al. 2006).

Whatever the origins of monandry, this mating system implies a limited availability of receptive females. The ability of males to find potential partners will thus depend on female distribution in space and time (Emlen and Oring 1977). The spatial distribution of females can be modulated by their degree of sociality: social behavior (aggregation) favoring mate encounters (e.g., *Blattella germanica*, Blattaria: Blattelliidae; Wileyto et al. 1984). For the temporal distribution of females, in insects, males often emerge before females (i.e., protandry; Morbey and Ydenberg 2001). Although its adaptive significance is still debated (Rhainds 2010), one hypothesis to explain protandry is the mating opportunities hypothesis, in which polygynous males gain in being mature before females, particularly if females mate only once during their lifetime and if opportunities for finding a mate are not limited (Zonneveld and Metz 1991). Protandry is also more susceptible to occur when mating with virgin females is advantageous for males (Wedell 1992).

**Figure 1:**
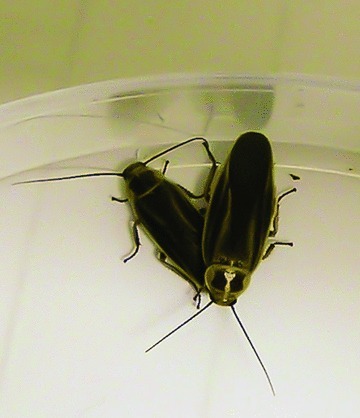
Male (left) and female (right) *Schultesia nitor*.

*Schultesia nitor* (Blaberidae: Zetoborinae, Grandcolas 1991; Fig. 1) is one of the two South American cockroach species belonging to the *Schultesia* genus with *S. lampyridiformis* (Roth 1973). In both cases, these species are strictly restricted to patchy habitats (Cacique bird’s—*Cacicus* and *Psarocolius* ssp.—pouch-like nests; Roth 1973; van Baaren et al. 2002). Larval development in this species is achieved in five to seven molts in males, and six to nine molts in females, supposing the occurrence of protandry. This is a gregarious cockroach: all individuals live in the same habitat and gregariousness tends to increase with age (Grandcolas 1993; van Baaren and Deleporte 2001; van Baaren et al. 2002, 2007). *S. nitor* females do not provide parental care to young nymphs as they disperse just after birth due to the mother’s aggressiveness (van Baaren et al. 2007) contrary to *N. cinerea* females which protect nymphs during their first instar (Moore and Moore 2001). Thus, maternal care is not a limiting factor for producing many broods during their lifetime, except if there is a refractory period (during pregnancy and after mating), a rather common phenomenon among cockroaches (Ringo 1996). Nevertheless, females produce only one brood during their life span. After nymphs are born, most females die within 15 days (J. van Baaren, unpubl. data).

Due to these specific characteristics, several predictions on *S. nitor* sexual behavior can be formulated. First, males are supposed to be mature before females and soon after their emergence. Thus, we determined sexual receptivity of both males and females *S. nitor* in the days following the imaginal molt to verify if protandry occurred. Second, we do not know if the single brood the females produced means that they mate only once or several times, and then store sperm from different males, which could potentially imply sperm competition (e.g., *N. cinerea*; Moore et al. 2001, 2003). To verify this prediction, we tested both males and females acceptance for remating. Third, if females were proved to mate only once (monandry), their ability to discriminate virgin (nondepleted males) from nonvirgin (depleted) males should be advantageous to maximize the number of fertilized eggs. Although gregariousness increases potential opportunities for mating, if female *S. nitor* proves to be monandrous, gregariousness would also increase male–male competition. Thus, we suspect the existence of male strategy for mating with virgin females. In the context of an unpredictable habitat, we expected that traits involved in *S. nitor* reproduction could differ from those exhibited by cockroach species living in more predictable habitats, like *N. cinerea*.

## Material and Methods

### Origin of the laboratory population

Individuals involved in the following experiments (realized in 2004) were caught in 1998 in French Guyana (Counamama, GPS: 05°27′11.6″ N 053°08′30.4″ W). They were reared in a laboratory at 26 ± 1C in 12L: 12D photoperiod in large boxes containing hundreds of individuals. Food (dry dog food) and water were given ad libitum.

### *Schultesia nitor* courtship behavior

In cockroaches, courtship behavior has been extensively described in different species (Roth and Willis 1952, 1954). According to preliminary observations, courtship behavior in *S. nitor* corresponds to this behavioral sequence exhibited by *B. germanica*, for example (Roth and Willis 1952). Male courtship behavior consists of wing raising to uncover tergal glands, facing away from the female’s head. The female mounts on the male’s back to lick tergal gland secretions while the male attempts to clasp the female’s genitalia. Once the genitalia are clasped, the male and the female stay end-to-end until copulation terminates. Copulation durations in *S. nitor* were about 2–3 h long (range 1–24 h).

### Experimental design

*Schultesia nitor* larval development is achieved in five to seven molts in males, and six to nine molts in females. Two instar nymphs were regularly collected from rearing boxes and isolated in sex-specific boxes (same conditions as rearing boxes) to avoid potential copulation. Every day, imagos were checked and isolated in sex- and age-specific boxes. Ages were therefore based on the delay after emergence (e.g., 0 for teneral individuals [newly emerged, not yet fully sclerotised and melanized], and 1 for adults having achieved their ecdysis the day before). As this species is more active at nightfall and does not detect red light (Barth 1964; Deleporte 1988), experiments were done using red light.

### Age of sexual maturity in males and females

To evaluate sexual maturity and variations in attractiveness, one virgin individual was tested with one virgin mature individual of the opposite sex in a petri dish (diameter = 140 mm, height = 20 mm) for 10 min. Four groups of individuals were tested: (1) consisted of female adults from teneral to 10 days old, paired with virgin males on average 7 days old (range 5–15 days); (2) male adults from 1 to 10 days old, paired with virgin females on average 7 days old (range 5–10 days); (3) adult females over 15 days old (range 15–21), paired with virgin males on average 7 days old (range 5-15 days); and (4) adult males over 15 day old (range 15–21), paired with virgin females on average 7 days old (range 5–10 days). For most of the trials, at least 10 pairs of different individuals were tested (see Table 1 for sample size).

**Table 1:** Trial summaries for virgin male and female *Schultesia nitor*

	Males				Females				
		
Age	*N*	Pdcb (±SE)	Cl (range)	Ncd (range)	*N*	Pdcb (±SE)	Pfma	Ml (range)	Nma
0	-	-	-	-	7	0.57 ± 0.18	0.50 ± 0.35	51–81	0
1	10	0.00 ± 0.00	-	-	21	0.29 ± 0.10	0.17 ± 0.38	318	0
2	10	0.80 ± 0.13	11–271	0–11	15	0.27 ± 0.11	0.00 ± 0.00	-	-
3	10	0.80 ± 0.13	26–311	0–28	13	0.08 ± 0.07	1.00 ± 0.00	120	-
4	10	0.80 ± 0.13	8–378	0–22	11	0.18 ± 0.12	0.50 ± 0.50	345	0–4
5	10	0.70 ± 0.14	17–42	0–24	11	0.45 ± 0.15	0.80 ± 0.20	16–224	0–9
6	10	0.80 ± 0.13	7–148	0–43	11	0.45 ± 0.15	0.60 ± 0.28	68–447	0–4
7	11	0.45 ± 0.15	18–173	0–31	14	0.57 ± 0.13	0.63 ± 0.22	39–368	0–4
8	11	0.82 ± 0.12	2–201	0–26	14	0.43 ± 0.13	0.50 ± 0.29	20–110	0–6
9	10	0.60 ± 0.15	0–158	0–40	11	0.54 ± 0.15	0.83 ± 0.17	68–390	0–1
10	10	0.60 ± 0.15	8–231	0–35	13	0.85 ± 0.10	0.91 ± 0.09	10–359	0–3
+15	9	0.89 ± 0.10	3–80	1–5	12	0.17 ± 0.11	1.00 ± 0.00	15–35	0

Age in days postemergence. *N*, total number of trials; Pdcb, proportion of trials where males displayed courtship behavior (±SE); Cl, courtship latency(s); Ncd, number of courtship displays; Pfma, proportion of females accepting to mate over trials with males displaying courtship behavior (±SE); Ml, mating latency (s); Nma, number of mating attempts.

To determine if the individuals being tested were sexually mature and attractive, different patterns were recorded according to the sex of the individual. For males, their capacity to produce courtship behavior was used to determine whether the individual was sexually mature or not. Copulation was not used here as it also depended on females. Other parameters were recorded to assess variation in attractiveness and motivation: (1) latency of courtship behavior (defined as the delay between the first contact and the first courtship behavior), and (2) number of courtship events. For females, we only considered trials in which males displayed courtship behavior. We only recorded the latency to mate.

### Capacity for multiple copulations

To evaluate the ability for multiple copulations for both sexes, mature virgin individuals (females five to 10 days old and males five to 15 days old) were presented to individuals which had previously mated (see Table 2 for details about sample size and age). We chose to test two delays after the first mating (1) 24 h to account for possible sperm depletion and (2) three weeks to be sure that males were sperm replenished. In the lobster cockroach (*N. cinerea*), for example, Montrose et al. (2004) demonstrated that males which had mated within 24 h were sperm depleted compared with those which had a five-day recovery time between two copulations and produced less offspring than virgin ones. For males, we recorded the proportion of males displaying courtship behavior. For females, we only recorded mating acceptance.

**Table 2:** Trial summaries for *Capacity to multiply copulations* experiment in male and female *Schultesia nitor*

Previously mated individuals		Virgin individuals					
					
Sex	Age	Sex	Age	*D*	*N*	Pdcb	Pfma
Males	5–15	Females	5–10	24 h	14	0.93 ± 0.07	0.92 ± 0.07
	4 weeks	Females	5–10	3 weeks	12	0.92 ± 0.08	1.00 ± 0.00
Females	5–10	Males	5–15	24 h	10	0.60 ± 0.15	0.00 ± 0.00
	4 weeks	Males	5–15	3 weeks	9	0.22 ± 0.14	0.00 ± 0.00

D, delay between first mating and trial (24 h or 3 weeks); *N*, total number of trials; Pdcb, proportion of trials where males displayed courtship behavior (±SE); Pfma, proportion of females accepting to mate over trials with males displaying courtship behavior (±SE).

### Discrimination between virgin and nonvirgin males

Because our experiment on the capacity of multiple copulations showed that females accepted to mate only once (see Results), the ability to discriminate between mated and unmated (virgin) males was tested by confronting virgin females with one mated and one virgin male. Three different series of trials involving one mature virgin female (five to 10 days old) and two males (five to 15 days old) were completed: (1) two virgin males (VV), (2) one virgin male and one previously mated within 24 h (VM), and (3) two previously mated males within 24 h (MM). One male per trial was marked (according to their status) allowing us to identify them during the experiment. Different parameters were recorded as follows: (1) which male met the female first to evaluate if encounter order could have an effect on mate choice, (2) which male displayed courtship behavior, (3) which male mated with the female, and (4) the number of courtship events.

### Statistical analyses

Chi-square tests (or Fisher’s exact test if sample size was below five individuals) were used to compare the proportion of: male courtship behavior and female mating between ages, female mating between the *Capacity for multiple copulations* and the *Determination of sexual maturity* experiment, successful males between both virgin and nonvirgin ones, and successful males between the first and the second ones to encounter the female. Binomial tests were used to compare the effect of encounter and courtship display order on mating success in the *Discrimination between virgin and nonvirgin males* experiment. Two-tailed nonparametric Wilcoxon rank-sum tests were used to compare latencies and the number of courtship events between two ages and linear regressions for variation among more than two consecutive ages. For the latter, variables were square root-transformed to respect the assumptions of the model. Medians were presented with their first and third inter-quartiles and proportions with their standard errors (se = √(*pq*/*n*); with *p* the proportion, *q* = 1 –*p* and *n* the sample size; Crawley 2007). All statistical treatments were done with R software (v. 2.10.1 R Development Core Team 2008) implemented with the *nlme* package for the linear regression.

## Results

### Age of sexual maturity in males and females

#### Male sexual maturity

Male courtship behavior depended on age (Chi-square test: 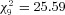, *P* < 0.01; Table 1), due to the fact that at day 1 after emergence, no male displayed courtship behavior contrary to the following days (Chi-square test: 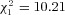, *P* < 0.01). For two- to 10-day-old males, the proportion of males displaying courtship behavior was homogenous (Chi-square test: 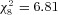, *P* = 0.56) with more than 45% of males displaying courtship behavior.

**Figure 2:**
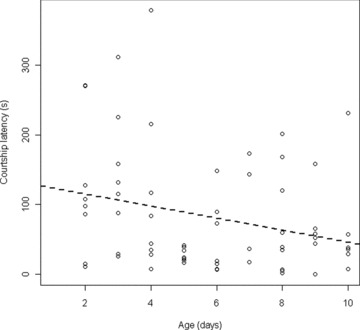
Variation in male courtship latencies according to their age. Dotted line represents linear regression.

A decrease in courtship latency was observed over the 10 days after emergence (Linear regression: *R*^2^ = 0.06, *F*_1,63_ = 5.40, *P* < 0.05; Fig. 2), but the overall number of displays did not vary (Linear regression: *R*^2^ = –0.003, *F*_1, 90_ = 0.67, *P* = 0.42). No difference between 10 and more than 15-day-old males was detected for the proportions of males displaying courtship behavior among trials (Chi-square test: 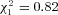, *P* = 0.36; Table 1), or in courtship latency or in number of display (Wilcoxon rank-sum test, respectively: *U* = 20.5, *N* = 73, *P* = 0.70; *U* = 38, *N* = 73, *P* = 0.89; Table 1). In summary, one-day-old males were not able to display courtship behavior. After the first day, courtship latency decreased but not the number of courtship displays.

#### Female sexual maturity

Overall, the proportion of females accepting to mate was homogenous (Chi-square test: 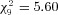, *P* = 0.78; Table 1). No variation in mating latency was observed (Linear regression: *R*^2^ = –0.01, *F*_1, 31_ = 0.55, *P* = 0.46). Teneral females did not mate significantly more than one-day-old females (Chi-square test: 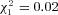, *P* = 0.88; Table 1). No difference was observed between 10- and 15-day-old females for mating (Chi-square test: 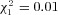, *P* = 0.90; Table 1). However, for the ones who accepted to mate, mating happened more quickly than in 10-day-old females (mating latencies—first < median < third quartile: 10-day-old females: 68 < 111.5 < 285.5 sec; 15-day-old females: 20 < 25 < 30 sec; Wilcoxon rank-sum test: *U* = 62, *N* = 35, *P* < 0.05). In conclusion, one- to 21-day-old females were able to mate, but the 15-day-old ones tended to mate more quickly than their younger counterparts.

### Capacity for multiple copulations

Males having previously mated within 24 h or three weeks were able to mate de novo in more than 80% of trials (Table 2). There was no difference in the proportion of males displaying courtship behavior between males mated within 24 h and males mated within three weeks (Chi-square test: 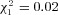, *P* = 0.89). In females, males displayed courtship behavior in only 60% of the trials (Table 2), but there was no difference between females mated within 24 h and females mated within three weeks (Chi-square test: 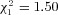, *P* = 0.22). Contrary to males, females never mated again (Table 2). Globally, male mating success was not affected by their mating status, and they were thus willing to multiply copulations even if they had already copulated with a female. Results of this experiment for females confirmed previous observations that they do not accept to mate more than once.

### Discrimination between virgin and nonvirgin males

Over the three different series of trials, not all the females accepted to mate (Table 3) but female acceptance for mating was equivalent in all trials (Chi-square test: 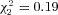, *P* = 0.91). However, in most trials, only one male displayed courtship behavior (Table 3). If we only considered trials in which both males displayed courtship behavior, the encounter order had no effect (Binomial test: VV, *P* = 0.12; MM, *P* = 0.62; and VM, *P* = 1) or globally (*P* = 0.09). In trials involving virgin against mated males (VM) with only one male displaying courtship behavior, 70% of them were virgin (Binomial test: *P* = 0.35) and most of trials were concluded by copulation without differences between virgin and mated males (Chi-square test: 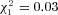, *P* = 0.86). In the case of both males displaying courtship behavior, females always mated with the virgin one (Fisher’s exact test: *P* < 0.05). Over the three series of trials, successful males did not display more courtship behavior than unsuccessful ones (Wilcoxon rank-sum test: *U* = 86, *N* = 28, *P* = 0.61). Virgin males displayed more courtship behavior than nonvirgin ones (courtship events—first < median < third quartile: virgin males: 3.25 < 5.00 < 7.75; nonvirgin males: 1.25 < 3.50 < 4.75; Wilcoxon rank-sum test: *U* = 54.50, *N* = 28, *P* < 0.05). In conclusion, encounter order was of no importance. Although results did not seem to indicate strong competition between males of different reproductive status, females preferred virgins over mated males.

**Table 3:** Trial summaries for *Competition between males* experiment in *Schultesia nitor*

Trials	*N*	Na	N_2_	Na_2_	First male mate
VV	11	0.91 ± 0.08	0.45 ± 0.15	0.80 ± 0.18	0.25 ± 0.22
VM	14	0.86 ± 0.09	0.29 ± 0.12	1.00 ± 0.00	0.50 ± 0.25
MM	13	0.92 ± 0.07	0.38 ± 0.13	1.00 ± 0.00	0.60 ± 0.22

Trials, two virgin males (VV), one virgin male and one previously mated (VM), and two previously mated males (MM); *N*, total number of trials; Na, proportion of trials with copulation (±SE); N_2_, proportion of trials where two males displayed courtship behavior (±SE); Na_2_, proportion of copulations in trials involving two males which displayed courtship behavior. First male mate: proportion of trials Na_2_ where the first male to encounter the female mate with it (±SE).

## Discussion

*Schultesia nitor* males began to display courtship behavior two days after their emergence. During the following days, courtship latency was stable but the number of courtship displays decreased. In females, tenerals were attractive and were able to mate with males. During the following couple of days, males were less attracted by females, but overall, females were receptive from their emergence to more than three weeks, but they never accepted to remate. Females over 15 days old accepted to mate more quickly than younger ones. Males were able to multiply copulations within 24 h, which indicated that there was no refractory period, or if so, it is less than 24 h. Males were also able to multiply copulation after three weeks of delay. When given the choice, females preferred virgin males and overall, virgin males were more successful at mating than nonvirgin ones.

### Capacity for multiple copulations: polygynous males and monandrous females, a classical scheme among cockroaches

As in most cockroach species (Bell et al. 2007), males *S. nitor* can remate once (present data) and up to 18 times (J. van Baaren, unpubl. data.) whereas females accepted to mate only once and became unreceptive after mating, excluding potential sperm competition. Cockroach mating systems have been studied regarding females’ sexual behavior, which displayed all possibilities from strict monandry to polyandry (Bell et al. 2007). Nevertheless, such classification is mainly the result of field or punctual observations rather than the results of sexual selection studies, limiting potential comparisons with others species. Principally two species have been well studied in a sexual selection context: *B. germanica* and *N. cinerea*. In both species, some females are able to remate, but most of them mate only once during their life (Cochran 1979; Schal et al. 1984; Moore and Moore 2001; Moore et al. 2003; Lihoreau and Rivault 2010). In *N. cinerea*, mechanical stimulus, due to the insertion of male spermatophore in female genitalia during copulation, inhibits female courtship feeding behavior which is an essential pattern of the mating process in cockroach species (Cornwell 1968; Roth 1969). Consequently, females become unreceptive (Roth 1962, 1964a) and regain receptivity after parturition for only one to two days (Roth 1962, 1964a,b). Female *S. nitor* were not receptive during their pregnancy. Although we cannot exclude that they regain receptivity after parturition, most of them die within a few days after their nymphs are born, limiting opportunities for remating and producing another clutch. Consequently, female *S. nitor* mate only once and are thus a monandrous species. Nonetheless, mechanisms leading to the absence of remating in *S. nitor* should be investigated, particularly to test if males are able to manipulate females. As a result of monandry, females have only one attempt to fertilize all of their ovocytes. Thus, their ability for discriminating between virgin and nonvirgin males to avoid potential sperm limitation is of particular interest.

### Discrimination between virgin and nonvirgin males: female preference versus male motivation?

In the experiments, females preferred virgin males. Nevertheless, as in most trials, only one male displayed courtship behavior, we could not establish a difference between active discrimination by the female, and male passivity due to potential hierarchical status. In *Gromphadorhina portentosa* (Blaberidae), dominant males are able to inhibit other male behavior resulting in dominance hierarchies with a wide range of aggressive behaviors (Barth 1968; Breed et al. 1981; Clark and Moore 1994; Clark 1998). Such social hierarchy has also been described in *N. cinerea*: dominant males being more susceptible to access virgin females than subordinate ones (Moore et al. 2001). However, no such hierarchical system has been yet described in *S. nitor*. Globally, virgin males were more motivated than nonvirgin ones. This difference may either be the result of a male mating strategy consisting in choosing to invest more in mating with the first potential mate they encounter (“random mating strategy”; see Bonduriansky 2001) or the physical incapacity of nonvirgin males to produce the same level of courting displays. In sagebrush cricket species *Cyphoderris strepitans* (Orthoptera: Haglidae), for example, nonvirgin males were not able to generate the same calling activity (used for attracting females) as virgin ones as a consequence of energy depletion during copulation due to nutrient investment in mating (Sakaluk et al. 1987; Sakaluk and Snedden 1990; Sakaluk and Ivy 1999). Both situations (mating strategy or energy depletion) would result in a higher level of success for virgin males independently of female mate preference. Moreover, previous studies on *B. germanica* have shown that male mate choice also has a role in mate selection, particularly for avoiding inbreeding in this mixed-family gregarious species (Lihoreau et al. 2008; Lihoreau and Rivault 2010). Thus, additional experiments investigating male mate choice should be considered to understand its relative importance in mating. Female mate preferences should also be further analyzed because mate choice can be based on several cues (Candolin 2003). In our study, we only discriminated males based on their mating status, but other cues could potentially be involved in female choice. We also observed that females over 15 days old accepted less courtship effort than their younger counterparts, which is similar to female *N. cinerea* (Moore and Moore 2001). This result could hint at a decrease of female choosiness (i.e., the effort that a female is ready to invest in assessing mates; Jennions and Petrie 1997) over time: costs associated with delaying reproduction could exceed costs of testing different males, and consequently decrease choosiness.

Although *S. nitor* mate only once, like *N. cinerea* and *B. germanica*, our experiments not only revealed that their maturation delay was shorter than in these species, but also that females accepted to mate soon after emergence at the teneral stage.

### Age of sexual maturity in males and females

#### Maturation delay: a particularity of S. nitor?

There was no maturation delay in females and less than two days of delay in males contrary to both *N. cinerea* and *B. germanica* (Moore and Moore 1988, 2001; Nojima et al. 1999a; Lihoreau and Rivault 2010). In *S. nitor*, gregariousness increased during larval development and adults live in groups in bird nests (Grandcolas 1993; van Baaren and Deleporte 2001; van Baaren et al. 2002, 2007). Contrary to *B. germanica* and *N. cinerea* which live in stable habitats (respectively, man-made habitats and forest floor leaf litter in Tanzania), the *S. nitor* habitat could be considered as being less stable, reducing food availability, mating opportunities, and/or survival. Unpredictable habitats constitute a strong abiotic selective pressure which is known to have consequences on different traits like behavior (Dubbert et al. 1998; Goldberg et al. 2001) or reproduction (Välimäki et al. 2006; Perfito et al. 2007). In zebra finches, *Taeniopygia guttata*, individuals living in an arid habitat with aperiodic unpredictable rainfalls maintain active reproductive systems contrary to those living in a predictable habitat (Perfito et al. 2007). In *P. napi*, females exhibit two strategies: they can either be monandrous (low mating rate, LMR) or polyandrous (high mating rates, HMR). Although HMR females have longer lifetime fecundity overall, LMR females have higher fitness gains at the beginning of their lifetime fecundity than HMR (Välimäki et al. 2006). Thus, if weather conditions changed rapidly LMR females are favored. In *S. nitor*, sexual life-history traits and surrounding physiological mechanisms could have been selected to balance costs generated by habitat instability, particularly reduced adult survival which is balanced by earlier reproduction attempts and faster development to reach maturity (Stearns 1976, 1989, 2000; Gasser et al. 2000; Haugen 2000; Braby 2002; Hernaman and Munday 2005). Although males observed a one-day delay before maturation whereas females did not, they experienced shorter larval development compensating for the adult maturation delay, thus making them available at female imaginal molt. As highlighted by Larsdotter Mellström et al. (2010), protandry is doubly beneficial for males: in maximizing mating opportunities, but also in allowing males to be mature at female emergence. In *S. nitor*, protandry should be of particular interest due to the fact that maturation delay in females is so reduced that they can mate at teneral stage.

#### Females mating as teneral

In females, postemergence attractiveness has been previously described in different species, due to female-like sex-appeal characteristic of exuvia products (Roth and Willis 1952; Schal and Bell 1983; Goudey-Perrière 1987). Teneral mating in cockroach species has been previously documented in two species belonging to the Blaberidae family (*Jagrehnia madecassa*, Sreng 1993; *Diploptera punctata*, Roth and Willis 1955), and one species belonging to the Blattidae family (*Neostylopyga rhombifolia*, Roth and Willis 1956). However, few studies concerning mating systems in these species are available, limiting potential comparisons with *S. nitor.* One noticeable exception, however, concerned *D. punctata* which, contrary to *S. nitor*, is viviparous. In this species, individuals produce secretions from defensive glands which are empty at the time of emergence. During the imaginal molt, females are doubly vulnerable until the full sclerotization of their body, and the replenishment of their defensive glands. Although males in *D. punctata* display mate guarding to monopolize female penultimate instar nymphs, as they benefit from this vulnerable stage (Schal et al. 1984), teneral mating also seems to provide indirect male protection to females (Roth and Willis 1955; Schal et al. 1984; Wyttenbach and Eisner 2001). Monandry limits male opportunities for mating and strategies enhancing probabilities of mating with virgin females should be favored. In *S. nitor*, teneral females accept to mate with males, produce broods (J. van Baaren, unpubl. data), and males obtain mating with a nonreluctant, virgin female. From the male point of view, such strategy suggests that they are able to monopolize females in their penultimate instar. Nevertheless, teneral mating also questions the implication of female mate choice. Obviously, during the teneral stage, they cannot avoid mating by flight. However, forced copulation cannot be involved in cockroaches as female mounting and feeding behavior are required for copulation (Roth and Barth 1964), but males can lure females. Indeed, tergal secretions have been shown to be constituted of several nonvolatile and volatile compounds (mainly oligosaccharides, phospholipids, cholesterol, and various amino acids Nojima et al. 1999a, b, 2002; Kugimiya et al. 2002, 2003a, b) which act as a strong dietary supplement. That is why they have been considered as a nuptial gift (nutrient transfer from male to female during courtship behavior and/or copulation; see Vahed 1998) even if this qualification could be questioned as enhancement of male mating success is not always supported (Mondet et al. 2008). Finally, teneral females could only be attracted by the nutritional bait of tergal secretion compounds and not directly by males. Thus, teneral mating could be the result of either the female’s acceptance for mating or male manipulation.

## Conclusions and Perspectives

Like in *N. cinerea* and *B. germanica*, female *S. nitor* are monandrous and males polyandrous. In males, protandry is not expressed through maturation delay but in the shorter larval development leading them to be available when females are sexually receptive at the teneral stage. Such reproductive traits have probably been selected to face an unpredictable environment, which potentially limit opportunities for mating. Teneral mating raises the existence of male mate guarding to monopolize females in their penultimate instar but questions the role of female. Altogether, our results indicate that monandry in *S. nitor* is the result of the short life-span fecundity of females. Females in *S. nitor* appear to be a limited resource for males, indicating that strong selective pressure must be exercised on males. Several points should be investigated, particularly concerning the fitness benefits of teneral mating in males and females and the involvement of female choice in such mating strategy. Although further work is required, in our opinion, this species provides an interesting model for understanding sexual conflicts. Thus, we are convinced of the interest in continuing to study of the sexual behavior of *S. nitor*.
